# Can fiberoptic bronchoscopy be applied to critically ill patients treated with noninvasive ventilation for acute respiratory distress syndrome? Prospective observational study

**DOI:** 10.1186/s12890-016-0236-y

**Published:** 2016-05-31

**Authors:** Pervin Korkmaz Ekren, Burcu Basarik Aydogan, Alev Gurgun, Mehmet Sezai Tasbakan, Feza Bacakoglu, Stefano Nava

**Affiliations:** Department of Chest Disease, Ege University Medical Faculty, Bornova, 35100 Izmir Turkey; Department of Specialistic, Diagnostic and Experimental Medicine, Respiratory and Critical Care, Sant’Orsola Malpighi Hospital, Alma Mater Studiorum, University of Bologna, Bologna, Italy

**Keywords:** Bronchoscopy, Noninvasive ventilation, Acute respiratory distress syndrome, Diagnosis, Safety

## Abstract

**Background:**

Noninvasive ventilation (NIV) is a cornerstone for the treatment of acute respiratory failure of various etiologies. Using NIV is discussed in mild-to-moderate acute respiratory distress syndrome (ARDS) patients (PaO_2_/FiO_2_ > 150). These patients often have comorbidities that increase the risk for bronchoscopy related complications. The primary outcome of this prospective observational study was to evaluate the feasibility, safety and contribution in diagnosis and/or modification of the ongoing treatment of fiberoptic bronchoscopy (FOB) in patients with ARDS treated with NIV.

**Methods:**

ARDS patients treated with NIV and who require FOB as the diagnostic or therapeutic procedure were included the study. Intensive care ventilators or other dedicated NIV ventilators were used. NIV was applied via simple oro-nasal mask or full-face mask. Pressure support or inspiratory positive airway pressure (IPAP), external positive end expiratory pressure (PEEP) or expiratory positive airway pressure (EPAP) levels were titrated to achieve an expiratory tidal volume of 8 to 10 ml/kg according to ideal body weight, SpO_2_ > 90 % and respiratory rate below 25/min.

**Results:**

Twenty eight subjects (mean age 63.3 ± 15.9 years, 15 men, 13 women, PaO_2_/FiO_2_ rate 145 ± 50.1 at admission) were included the study. Overall the procedure was well tolerated with only 5 (17.9 %) patients showing minor complications. There was no impairment in arterial blood gas and cardiopulmonary parameters after FOB. PaO_2_/FiO_2_ rate increased from 132.2 ± 49.8 to 172.9 ± 63.2 (*p* = 0.001). No patient was intubated within 2 h after the bronchoscopy. 10.7, 32.1 and 39.3 % of the patients required invasive mechanical ventilation after 8 h, 24 h and 48 h, respectively. Bronchoscopy provided diagnosis in 27 (96.4 %) patients. Appropriate treatment was decided according to the results of the bronchoscopic sampling in 20 (71.4 %) patients.

**Conclusion:**

FOB under NIV could be considered as a feasible tool for diagnosis and guide for treatment of patients with ARDS treated via NIV in intensive care units. However, FOB-correlated life-treathening complications in severe hypoxemia should not be forgotten. Furthermore, further controlled studies involving a larger series of homogeneous ARDS patients undergoing FOB under NIV are needed to confirm these preliminary findings.

## Background

Noninvasive ventilation (NIV) is defined as any form of ventilatory support applied without endotracheal intubation [1]. Application of NIV is a cornerstone for the treatment of acute respiratory failure of various etiologies [2]. It reduces intubation rate in patients with exacerbations of chronic obstructive pulmonary disease (COPD) and acute cardiogenic pulmonary edema [3] and in immunocompromised patients with hypoxemic respiratory failure [4, 5]. Using NIV is discussed in mild-to-moderate acute respiratory distress syndrome (ARDS) patients (PaO_2_/FiO_2_ > 150) [6].

Fiberoptic bronchoscopy (FOB) may be required in some patients with acute respiratory failure in intensive care units (ICU), mainly as diagnostic tool or to remove abundant secretions [7, 8]. As a matter of fact it may also be applied to determine the cause of diffuse pulmonary infiltrates (infection, diffuse alveolar hemorrhage, organizing pneumonia) [9]. Other indications for FOB in critically ill patients consist of atelectasis, hemoptysis and suspicion of lung neoplasia. Patients in ICU for acute respiratory failure often have comorbidities that increase the risk of bronchoscopy related complications. Feasibility of bronchoscopy during NIV in patients with respiratory failure was shown previously and NIV was found to be superior to conventional oxygen supplementation for preventing gas-exchange deterioration during FOB [10, 11]. In those studies, NIV was used to facilitate bronchoscopy and NIV was not required prior to bronchoscopy. However, there is limited data on the feasibility and usefulness of FOB in patients who are already treated with NIV for acute respiratory failure [12] and in particular there is not enough evidence for using NIV in patients with ARDS [6]. Therefore, we aimed to evaluate the feasibility and safety of FOB in patients with ARDS ventilated with NIV and its contribution in the diagnosis and/or modification of the ongoing therapy.

## Methods

The patients hospitalized in 8-bed ICU at Ege University Medical School Department of Chest Diseases were assessed in terms of ARDS, NIV treatment and FOB application. The decision both to initiate NIV and to perform FOB were made by the pulmonologist. This prospective observational study was approved by the Ege University Ethical and Research Project Committee (2010-TIP-088). All study participants or legal representatives provided written informed consent before the procedures.

### Study population

The study population was obtained from the specified respiratory intensive care unit (January 2010-December 2014).

Inclusion criteria were: 1. ARDS diagnosed by Berlin criteria or The American-European Consensus Conference [13, 14], 2. Age ≥ 18 years, 3. Treated with NIV before the bronchoscopy 4. Requiring FOB with or without bronchoalveolar lavage (BAL) for diagnostic or therapeutic apporach, 5. Informed consent. Exclusion criteria were: 1. Refusal of NIV, 2. Presence of contraindications for NIV such as facial deformity, upper gastrointestinal bleeding, upper airway obstruction, inability to protect the airway, significantly altered mental status, severe haemodynamic instability, respiratory or cardiac arrest and acute coronary syndromes, 3. Tracheostomy or intubation before admission, 4. Presence of contraindications for bronchoscopy procedure such as insufficient platelet number (<70,000 cells/μL) and coagulation disorders, 5. Patients with NIV use at home prior to ICU admission.

Demographic characteristics, comorbid diseases, presence of immunosuppression, Acute Physiology and Chronic Health Evaluation II (APACHE II) score [15] and radiologic and laboratory findings were recorded for each patient. Acquired immunodeficiency syndrome, immunosuppressive medication, organ transplantation, high dose chemotherapy during the past 60 days, absolute neutrophil count < 1000/mL were defined as immunosuppression. Standard microbiological diagnostic measures before FOB and additional serological studies were ordered in immunosuppressed patients as indicated. Heart and respiratory rates, blood pressures and arterial blood gases were assessed 15 min before and 1 h after the FOB for this study. These measurements and ventilator settings were monitored during FOB.

### Noninvasive ventilation

**Fig. 1:**
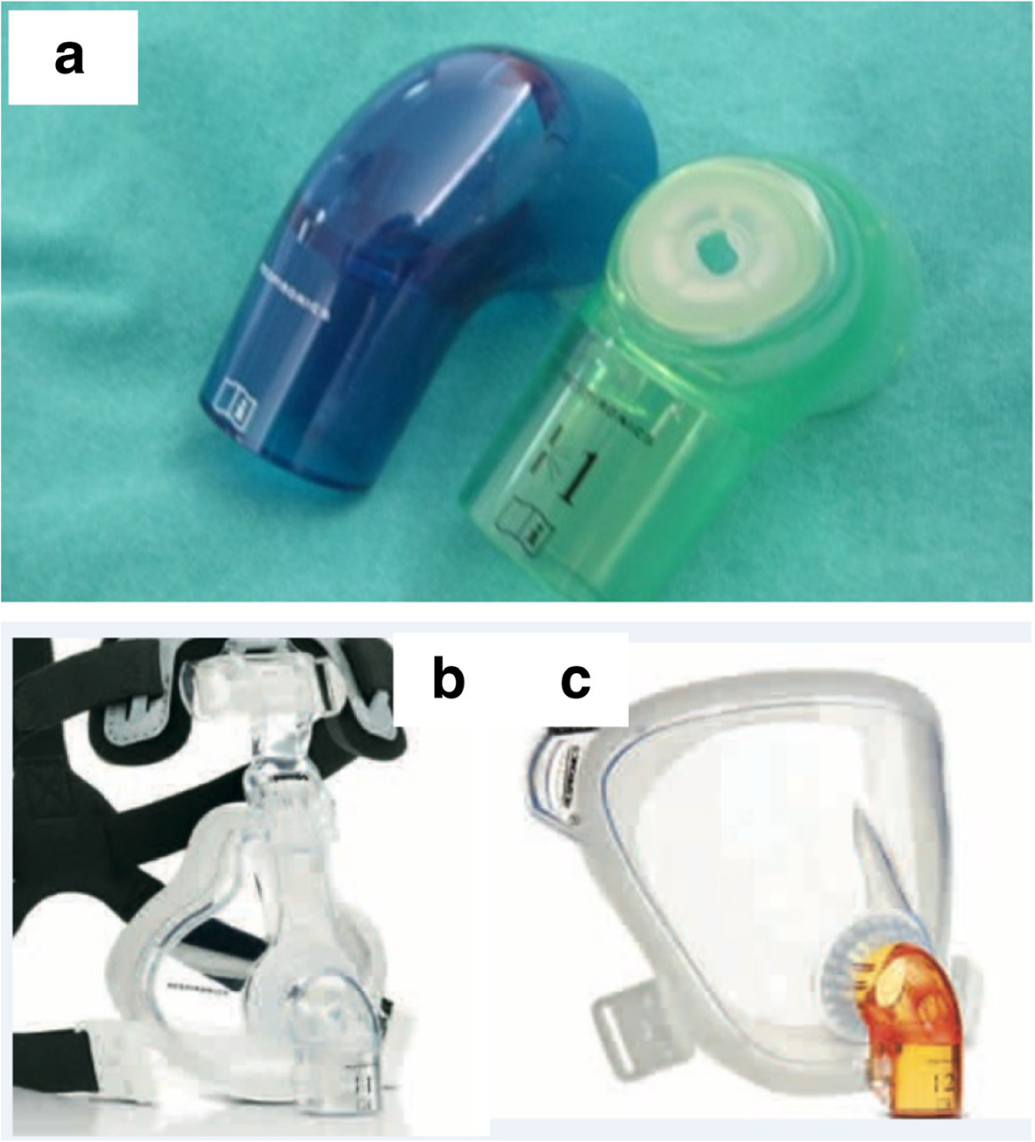
**a** Used connector between the ventilator tubing and the mask for the insertion of FOB, **b** Oro-nasal mask, **c** Full-face mask

All patients were on NIV prior and throughout the bronchoscopic sampling in the ICU. NIV was applied via simple oro-nasal mask (AF 531 Oro-nasal mask, Philips Respironics) or full-face mask (PerforMax Total face mask, Philips Respironics) (Fig. 1) and the ventilator settings were adjusted by the pulmonologist. ICU ventilator (Servo 900C, Siemens) or dedicated NIV ventilator (VIVO 40, General Electric) devices were set with the optimal required FiO_2_. Pressure support or inspiratory positive airway pressure (IPAP), external positive end expiratory pressure (PEEP) or expiratory positive airway pressure (EPAP) levels were titrated to achieve an expiratory tidal volume of 8 to 10 ml/kg according to ideal body weight, adjusted to maintain SpO_2_ > 90 % and respiratory rate below 25/min.

### Bronchoscopy procedure

Throughout the NIV treatment and bronchoscopy procedure electrocardiography, intra arterial blood pressure, pulse oximeter and ventilator parameters were monitored continuously. The ICU bronchoscopy team was composed of two well-trained pulmonologists (FB, MST) and two nurses. All bronchoscopies were performed while patients were in upright position and in their own beds. 10 % lidocaine spray solution and 2 % lidocaine solution were used for topical anaesthesia of nasopharynx and tracheobronchial tree, respectively. A connector (bronchoscopy elbow, Philips Respironics) was used between the ventilator tubing and the mask for the insertion of FOB (Olympus BFU 180F) (Fig. 1). Fiberoptic bronchoscopy was performed via nasal route. Short term sedation was administered in some patients according to their respiratory and hemodynamic situations. Sedation was started using 2 mg midazolam at the beginning of the procedure. The patients who did not achieve stabilisation received repetitive applications of 1 to 2 mg midazolam. Maximum total dose of midazolam was 5 mg. Patients were supplied with the necessary FiO_2_ which provided SpO_2_ > 90 % during FOB procedure (one or more of these procedures were performed in same FOB application: BAL, bronchoscopic brushings and biopsy, aspiration) under NIV. The bronchoscopic samples were sent to microbiology and pathology laboratories. Antimicrobial treatment was started empirically while the sampling was performed and, later treatment was adjusted according to microbiological results.

### Outcomes

Primary outcomes were: 1. Safety with the recording of any complications [such as fever, arrhythmia, hypoxemia (SpO_2_ < 90 %), hypotension (systolic and diastolic blood pressure below 90 mmHg and 60 mmHg, respectively), haemorrhage and pneumothorax]. 2. Contribution of FOB in diagnosis and/or modification of the ongoing treatment. 3. Requirement for endotracheal intubation was specifically observed within two hours and eight hours after the FOB. Criteria of two and eight hour limit for endotracheal intubation were adopted from previous studies [16, 17].

### Data analyses

Data management and analysis of all data were conducted via SPSS for Windows 16.0 software (SPSS, version 16, Chicago). Kolmogorov-Smirnov test was used to present the distribution of all recorded variables. Arterial blood gases and haemodynamic parameters before and after FOB were compared with Wilcoxon test. Statistical significance level was set at *p* < 0.05.

## Results

### Patient characteristics

Twenty eight subjects (mean age 63.3 ± 15.9 years, 15 men, 13 women) were included the study. 20 patients (71.4 %) had immunosuppression. Patients had hematologic malignancy, solid-organ tumor, renal transplantation, long-term corticosteroid treatment due to interstitial lung disease or connective tissue disease that caused immunosuppression. The most common admission diagnosis to ICU was pneumonia (Table 1). As shown on Table 2, FOB indications were: suspicion of opportunistic infections in 18 patients (64.3 %), alveolar hemorrhage in 4 patients (14.3 %), suspicion of malignancy in 4 patients (14.3 %), aspiration as therapeutic approach in 4 patients (14.3 %) and other indications were exacerbation of idiopathic pulmonary fibrosis, drug induced pulmonary toxicity and pulmonary involvement of rheumatoid arthritis. Fiberoptic bronchoscopy was performed with ICU ventilator in 82.1 % of patients and dedicated NIV ventilators were used for the remaining patients. PaO_2_/FiO_2_ ratio before FOB application was calculated as 132.2 ± 49.8. Twenty three (82.1 %) patients received 100 % FiO_2_ during FOB to allow SpO_2_ levels which must be achieved above 90 %. Pressure support, IPAP and EPAP/PEEP values during FOB were 9 (0–18), 20 (8–28) and 8 (5–12) cmH_2_O, respectively.

**Table 1:** Patient characteristics at admission (*n* = 28)

Age (yrs)	63.3 ± 15.9
Gender (male), *n* (%)	15 (53.6)
Comorbid illnesses, *n* (%) Coronary artery disease Hematological malignancy Interstitial lung disease Connective tissue diseases Chronic lung disease Solid organ malignancy Diabetes mellitus Chronic renal failure Others	27 (96.4) 9 (32.1) 7 (25.0) 6 (21.4) 6 (21.4) 5 (17.9) 4 (14.3) 3 (10.7) 3 (10.7) 3 (10.7)
Immunosuppression, *n* (%)	20 (71.4)
APACHE II score^a^	15.5 (9–27)
PaO_2_/FiO_2_	145 ± 50.1
The diagnosis of hospitalisation, *n* (%)	
Pneumonia Alveoler hemorrhage Respiratory failure (other etiology)	20 (71.4) 3 (10.7) 5 (17.9)
ARDS, *n* (%)	
Mild Moderate Severe	5 (17.9) 18 (64.2) 5 (17.9)
Chest X-ray findings, *n* (%)	
Bilateral alveolar infiltrates Bilateral interstitial infiltrates Bilateral interstitial and alveolar infiltrates	17 (60.7) 7 (25.0) 4 (14.3)

^a^Median value (range)

*Abbreviations: APACHE II* acute physiology assessment and chronic health evaluation II, *ARDS* acute respiratory distress syndrome

**Table 2:** Patients characteristics, indications and results of FOB

No	Diagnosis at hospitalisation	Immuno-suppression	PreFOB ARDS category	FOB indications	FOB diagnosis	Infectious agents	Intubation	Intubation time (hour)
1	Pneumonia	+	Severe	Opportunistic infections	Infection	CMV	-	
2	Pneumonia	+	Mild	Opportunistic infections	Infection	*Geotrichum capitatum*	+	24–48
3	Pneumonia	-	Severe	Opportunistic infections	Infection	*Aspergillus* spp., Respiratory virus	-	
4	Pneumonia	+	Mild	Opportunistic infections	Infection	*C. albicans*, CMV, Respiratory virus	-	
5	Pneumonia	+	Moderate	Opportunistic infections	Infection	*C. albicans*, CMV	-	
6	Alveoler hemorrhage	+	Moderate	Alveoler hemorrhage	Alveoler hemorrhage		-	
7	Respiratory failure	-	Moderate	Aspiration	Infection	Non-albicans *Candida*	+	>48
8	Pneumonia	+	Severe	Alveoler hemorrhage	Alveoler hemorrhage	CMV	+	8–24
9	Respiratory failure	-	Moderate	Aspiration	Infection	CMV, *Aspergillus* spp., *C. albicans*	+	>48
10	Pneumonia	-	Moderate	Malignancy	Infection	*K. pneumoniae*, CMV	-	
11	Pneumonia	-	Mild	Malignancy	Infection	*C. albicans*	+	8–24
12	Pneumonia	+	Moderate	Opportunistic infections	Infection	CMV, PcP	+	<8
13	Pneumonia	-	Severe	Opportunistic infections	Infection	CMV, *C. albicans*	-	
14	Pneumonia	-	Severe	Aspiration	Infection	*C. albicans*	+	8–24
15	Alveoler hemorrhage	+	Moderate	Alveolar hemorrhage	Infection	*C. albicans*	-	
16	Respiratory failure	+	Moderate	Opportunistic infections	Infection	CMV, PcP	+	<8
17	Pneumonia	+	Moderate	Opportunistic infections	Alveoler hemorrhage		-	
18	Respiratory failure	+	Mild	Opportunistic infections	Infection	*C. albicans*	+	24–48
19	Pneumonia	+	Moderate	Opportunistic infections	Malignancy Infection	*P. aeruginosa*	+	8–24
20	Respiratory failure	+	Severe	Opportunistic infections	Infection	*C. albicans*, Respiratory viruses	-	
21	Pneumonia	-	Moderate	Aspiration	-		+	8–24
22	Alveoler hemorrhage	+	Severe	Alveoler hemorrhage	Alveoler hemorrhage		-	
23	Pneumonia	+	Severe	Multiple etiologies^a^	Infection	*Acremonium*, CMV	+	<8
24	Pneumonia	+	Moderate	Multiple etiologies^b^	Infection	*C. albicans*, Respiratory viruses, CMV	+	>48
25	Pneumonia	+	Severe	Multiple etiologies^c^	Infection	*Acremonium*, *Aspergillus* spp., Respiratory viruses, CMV	+	>48
26	Pneumonia	+	Moderate	Opportunistic infections	Infection	*C. albicans*	+	8–24
27	Pneumonia	+	Severe	Multiple etiologies^d^	Malignancy		+	>48
28	Pneumonia	+	Moderate	Opportunistic infections	Infection	*M. tuberculosis*	+	>48

*Abbrevations: CMV* cytomegalovirus, *PcP Pneumocystis jiroveci* pneumonia

^a^Opportunistc infections, exacerbation of idiopathic pulmonary fibrosis

^b^Malignancy, alveoler hemorrhage, opportunistic infections, drug induced pulmonary toxicity

^c^Pulmonary involvement of rheumatoid arthritis, opportunistic infections, drug induced pulmonary toxicity

^d^Opportunistc infections, malignancy

### Tolerance and safety of fiberoptic bronchoscopy

The median duration of NIV prior to FOB was 3 h (range 0–72 h). Vasopressor/inotropic agents before the FOB application were begun in 4 patients (14.3 %) due to hemodynamic instability. Thirteen patients (46.4 %) received sedation for NIV and/or FOB. Maximum duration of the bronchoscopy was 20 min and FOB application was performed only once for each patients. Arterial blood gas analyses, PaO_2_/FiO_2_ rates and cardiorespiratory parameters before and after FOB were shown in Table 3. There was no impairment in these parameters after bronchoscopy. PaO_2_/FiO_2_ rate increased from 132.2 ± 49.8 to 172.9 ± 63.2 after procedure. Pressure support, IPAP, EPAP/PEEP and FiO_2_ levels did not change during FOB.

**Table 3:** Hemodynamic and arterial blood gas parameters before and after FOB

Parameters	Before FOB	After FOB	*p* value
Arterial blood gas			
pH	7.45 ± 0.12	7.36 ± 0.14	0.07
PaCO_2_ (mmHg)	41.3 ± 15.6	41.7 ± 16.1	0.56
PaO_2_/FiO_2_	132.2 ± 49.8	172.9 ± 63.2	**0.001**
Mean arterial pressure (mmHg)	96.7 ± 18.8	94.3 ± 19.6	0.43
Heart rate/min	104.0 ± 20.1	102.1 ± 18.6	0.48

The number in boldface reflects *p* < 0.05

Respiratory and hemodynamic variables were followed up very closely for the required intubation. Complications other than endotracheal intubation developed in 5 (17.9 %) patients within 12 h after FOB. Fever (*n* = 2), hypoxemia (*n* = 1) and hypotension (*n* = 2) occurred as complication of bronchoscopy. In one of these patients, hypotension developed within the first four hours. No other adverse effects were observed such as haemorrhage and pneumothorax.

Endotracheal intubation was not required within 2 h after the bronchoscopy. Intubation was performed only in three patients (10.7 %) within 8 h. Two of them had severe coinfection with *Cytomegalovirus* and *Pneumocystis jiroveci*. 32.1 and 39.3 % of the patients required IMV within 24 h and 48 h, respectively. Due to deterioration of respiratory failure or septic shock, 6 patients (21.4 %) were intubated after 48 h of the procedure. Five of them had hospital acquired infection in their follow ups.

### Clinical usefulness of fiberoptic bronchoscopy

Bronchoscopy contributed to diagnosis of 27 (96.4 %) patients. Bacteria (*n* = 2), fungus (*n* = 18), cytomegalovirus (*n* = 12), other viruses (*n* = 5) and *M. tuberculosis* (*n* = 1) were isolated in 23 (82.1 %) patients as underlying infectious agent. *Candida* spp. was accepted as cause of infection for 6 patients, because 3 patients were immunosuppressive and we found no other etiologic opportunistic infection agents in the remaining patients. Various combinations of the infectious agents were shown in 12 (42.9 %) patients. Alveolar hemorrhage was diagnosed in 4 patients and malignancy was reported as a result of cytologic examination in 2 patients. While secretions were being removed in 3 patients, atelectasis was resolved in one patient by FOB. Appropriate treatment was arranged in 20 patients (71.4 %) according to FOB results. Initial empirical treatments were appropriate in the remaining patients.

## Discussion

The study demonstrated that bronchoscopy under NIV is feasible, safe and an effective diagnostic procedure, therapeutic approach and guide for treatment in ARDS patients. After the procedure, arterial blood gas values, PaO_2_/FiO_2_ rate, and cardiac parameters did not change and none of the patients needed intubation within two hours after bronchoscopy. Fiberoptic bronchoscopy can be performed safely without major complications in this group of patients.

Diagnostic or therapeutic bronchoscopy is necessary for some ICU patients treated for acute respiratory failure, however it can be associated with various complications as well as mortality. Because of these risks, FOB can be challenging in these patients. Bronchoscope covers 10–15 % of the tracheal lumen and may increase respiration efforts and decrease PaO_2_ by 10–20 mmHg causing respiratory and cardiac complications [18]. Additionally, BAL sampling may cause worsening of oxygen desaturation [19]. The American Thoracic Society recommends avoiding FOB and BAL in patients with hypoxemia (PaO_2_ < 75 mmHg or oxygen saturation <90 % with supplemental oxygen) [20]. Although alternative approaches in these patients with higher-risk are empirical treatments or intubation for FOB, the observational study and the randomized controlled trials have shown that NIV may be an alternative to endotracheal intubation in these critically ill patients who are not previously ventilated [10, 11, 21].

Early etiological diagnosis in critically ill patients is important for appropriate treatment. Baumann et al. [12] showed that BAL during NIV yielded diagnostic information in 68 % of the patients with acute respiratory failure. Diagnostic success of FOB was 59 % in another study in which oxygen supplementation or NIV application was used during the procedure [22]. Our immunosuppressed patient ratio was higher than the study population of Bauman et al. and Cracco et al. (71.4 % vs 53 %, 53 %, respectively). Clouzeau et al. [16] found that diagnostic or therapeutic information ratio was 75 % in patients who underwent FOB under NIV. 83.3 % of their patients were immunocompromise. In our study, diagnostic accuracy of FOB in ARDS patients was 96.4 %. High diagnostic rate in our study can be interpreted with the fact that we performed sampling with FOB just as the patients apply to ICU. At the same time, FOB application allowed the therapeutic approaches such as aspiration. Furthermore, Agarwal et al. [23] have shown that NIV-assisted bronchoscopic lung biopsy is another diagnostic method in hypoxemic patients with diffuse lung infiltrates.

Azoulay et al. [24] found that deterioration in respiratory status occurred in 44.8 % of patients who had BAL with oxygen. But only 18.7 % of their patients underwent BAL with NIV. Chiner et al. [21] showed that there were no significant differences in arterial blood gas levels in patients with acute respiratory failure before FOB and 2 h after FOB. Another study showed a significant improvement in arterial blood gas levels of patients with acute decompensated COPD due to community-acquired pneumonia second hour after FOB with NIV [25]. Secretion aspiration might lead to improvement. In our study, there were no significant changes in arterial blood gas analyses and cardiac parameters after FOB. Other complications such as cardiac arrhythmia, pneumothorax or hemoptysis did not occur in our study population.

Only three patients (10.7 %) were intubated within 8 h after FOB (5 h, 7 h and 8 h, respectively). Underlying diseases of these patients were interstitial lung disease and lung involvement due to connective tissue disease. The patients were receiving systemic corticosteroid therapy before admission to hospital and FOB was applied to diagnose opportunistic infections. Three patients died within 24 h after the procedure. FOB should not be considered in these cases, because they had severe underlying diseases and infections when they were admitted. Identified mixt infectious agents had a major effect in their fast progression. *Cytomegalovirus* and *Pneumocystis jiroveci* in two patients, *Acremonium* and *Cytomegalovirus* in another patient were shown as opportunistic infectious agents. Fiberoptic bronchoscopy usually causes alteration in gas exchange. In hypoxemic intubated patients, PaO_2_ returns to baseline within 2 h [26]. None of the patients in the current study needed intubation within 2 h after FOB. Based on this information, FOB may not have led to intubation in three patients. Baumann et al. [12] found that 40 patients required NIV prior to the decision to use FOB. Four of them (10 %) required IMV within 8 h. Within 48 h, 45 % of their patients were intubated. In Baumann et al.’s study, mean baseline PaO_2_/FiO_2_ value was 176 ± 54 which is higher than the values of the current study. In the current study 10.7, 32.1 and 39.3 % of patients required IMV within 8 h, 24 h and 48 h, respectively. Clouzeau et al. [16] assessed BAL application under NIV in which PaO_2_/FiO_2_ of their patients was 181 ± 50 before FOB. None of the patients was intubated within 2 h, four of 23 patients (17.4 %) were required IMV within 24 h after the procedure. Presence of the COPD and immunosuppression were shown as risk factors for intubation [22]. Intubation rate in the current study is acceptable according to presented rates in the literature. Intubation rate reached to 54 % in patients with acute respiratory failure who received NIV. Also, NIV failure ratio was 62 % in moderate and 84 % in severe ARDS [27]. Cases in the current study were not intubated due to sudden gas exchanges and hemodynamic deterioration after bronchoscopy. Severe underlying diseases and infection situations should be taken into consideration for IMV.

The authors of this study aware of the discussions about using NIV in patients with ARDS. But a recent study which assessed intubation rate and risk factors of NIV failure in patients with non-hypercapnic acute hypoxemic respiratory failure showed significant differences for intubation rate among four groups (non-ARDS, mild ARDS, moderate ARDS, severe ARDS). However, the mortality rate was not statistically significant and the time to intubation had no effect on patient mortality [27]. In patients with hematologic malignancies and acute respiratory failure, delayed (after NIV failure) vs. immediate IMV was associated with increased hospital mortality which was not statistically significant (65 % vs. 58 %) [28]. Early diagnosis and beginning time of true treatment in patients with immunosuppression may be as important as ARDS severity. The authors think that FOB under NIV treatment due to ARDS may be applied to patients whose PaO_2_/FiO_2_ ratio is close to 100 when their hemodynamic status is stable and procedure is performed in ICU where all intubation equipment for emergency intubation exist.

This study has some limitations. First, there are no control groups. Enrolment of a control group was not possible due to ethical reasons, because treatment with NIV requires in patients with acute respiratory failure. In other words, it is believed that intubation is not advisable solely for FOB applications. Second, the study population in the current study was small. Third, the study population was composed of patients with hypoxemic and hypercapnic respiratory failure. Fourth, the data about excluded patients was not given and limited number arterial blood gas analyses were assessed after FOB. Fifth, these results were from a single center.

## Conclusions

In conclusion, FOB under NIV may be safely and effectively applied for diagnosis and treatment of patients with ARDS in ICU units that is adequately equipped and staffed for intubation. There are high risks associated with intubation during FOB and after procedure. Therefore, these patients should be closely followed up for ventilation and for other vital parameters. FOB with NIV may be considered in patients with ARDS for diagnosis and treatment, so intubation and its complications may be avoided in this patient group. More studies with large sample sizes are required to make a more thorough assessment of FOB application under NIV in patients with ARDS.

## Abbreviations

APACHE II: Acute Physiology and Chronic Health Evaluation II
ARDS: acute respiratory distress syndrome
BAL: bronchoalveolar lavage
COPD: chronic obstructive pulmonary disease
EPAP: expiratory positive airway pressures
FOB: fiberoptic bronchoscopy
ICU: intensive care unit
IPAP: inspiratory positive airway pressure
NIV: noninvasive ventilation
PEEP: positive end expiratory pressure
